# Inhibition of KDM5A attenuates cisplatin-induced hearing loss via regulation of the MAPK/AKT pathway

**DOI:** 10.1007/s00018-022-04565-y

**Published:** 2022-11-17

**Authors:** Chang Liu, Zhiwei Zheng, Wen Li, Dongmei Tang, Liping Zhao, Yingzi He, Huawei Li

**Affiliations:** 1 Department of ENT Institute and Otorhinolaryngology, Eye and ENT Hospital, State Key Laboratory of Medical Neurobiology and MOE Frontiers Center for Brain Science, Fudan University, 83 Fenyang Road, Shanghai, 200031 China; 2 NHC Key Laboratory of Hearing Medicine, Fudan University, Shanghai, 200031 People’s Republic of China; 3 Institutes of Biomedical Sciences, Fudan University, Shanghai, 200032 People’s Republic of China; 4 The Institutes of Brain Science and the Collaborative Innovation Center for Brain Science, Fudan University, Shanghai, 200032 People’s Republic of China

**Keywords:** KDM5A, Hair cells, Cisplatin, Ototoxicity, Apoptosis

## Abstract

The study aimed to investigate the potential role of lysine-specific demethylase 5A (KDM5A) in cisplatin-induced ototoxicity. The effect of the KDM5A inhibitor CPI-455 was assessed by apoptosis assay, immunofluorescence, flow cytometry, seahorse respirometry assay, and auditory brainstem response test. RNA sequencing, qRT-PCR, and CUT&Tag assays were used to explore the mechanism underlying CPI-455-induced protection. Our results demonstrated that the expression of KDM5A was increased in cisplatin-injured cochlear hair cells compared with controls. CPI-455 treatment markedly declined KDM5A and elevated H3K4 trimethylation levels in cisplatin-injured cochlear hair cells. Moreover, CPI-455 effectively prevented the death of hair cells and spiral ganglion neurons and increased the number of ribbon synapses in a cisplatin-induced ototoxicity mouse model both in vitro and in vivo. In HEI-OC1 cells, KDM5A knockdown reduced reactive oxygen species accumulation and improved mitochondrial membrane potential and oxidative phosphorylation under cisplatin-induced stress. Mechanistically, through transcriptomics and epigenomics analyses, a set of apoptosis-related genes, including *Sos1*, *Sos2*, and *Map3k3*, were regulated by CPI-455. Altogether, our findings indicate that inhibition of KDM5A may represent an effective epigenetic therapeutic target for preventing cisplatin-induced hearing loss.

**Supplementary Information:**

The online version contains supplementary material available at 10.1007/s00018-022-04565-y.

## Background

Cisplatin (CP) is a widely used agent for the treatment of various malignant cancers, including head and neck cancers, breast cancers, and lung cancers [1]. However, its application is seriously limited due to adverse effects [2, 3]. Ototoxicity is a severe side effect characterized by irreversible, progressive, and bilateral hearing loss [4]. Despite years of research, the underlying mechanism of CP-induced ototoxicity remains unclear, and little is known about the potential regulators in this process of ototoxicity.

Small molecules that interfere with protein–protein or protein–DNA interactions have offered a promising approach to controlling gene regulation. For example, gastrin-releasing peptide receptor (GRPR) acts as an inflammatory factor, and GRPR antagonists induce senescence and antiproliferative effects in several cancers [5]. Nickols et al. [6] revealed that targeting hypoxia-inducible factor (HIF-1)-DNA binding might be clinically relevant due to its complex role in cell survival and death pathways [7]. In addition, several studies focused on the development of novel treatment options [8], such as echinococcal infection [9] and chitosan–tripolyphosphate nanoparticles [10], as promising approaches in various cell lines. In recent years, the role of epigenetic modifications in the auditory field has received widespread attention [11–15].

Epigenetics refers to reversible and hereditary changes in gene function without altering the DNA sequence of the nucleus. Histone methylation is one of the most deeply studied post-translational modifications which regulate many biochemical processes [16]. As a member of the family of Jumonji C (JmjC) domain-containing histone demethylases, KDM5A regulates the demethylation of H3K4me3, an epigenetic mark associated with transcriptional activation [17]. Mounting evidence suggests that KDM5A plays an essential role in mitochondrial metabolism [18], cell cycle progression [19], cellular senescence [20], development and differentiation [21], and tumorigenesis [22]. However, the role of KDM5A in CP-induced ototoxicity is poorly understood. Here, we demonstrated that inhibiting KDM5A activity with CPI-455 reduces mitochondrial apoptosis, thus broadening our understanding of epigenetic modifications as novel targets to prevent CP-induced hearing loss.

## Results

### CPI-455 protects cochlear hair cells (HCs) against CP ototoxicity in vitro

**Fig. 1:**
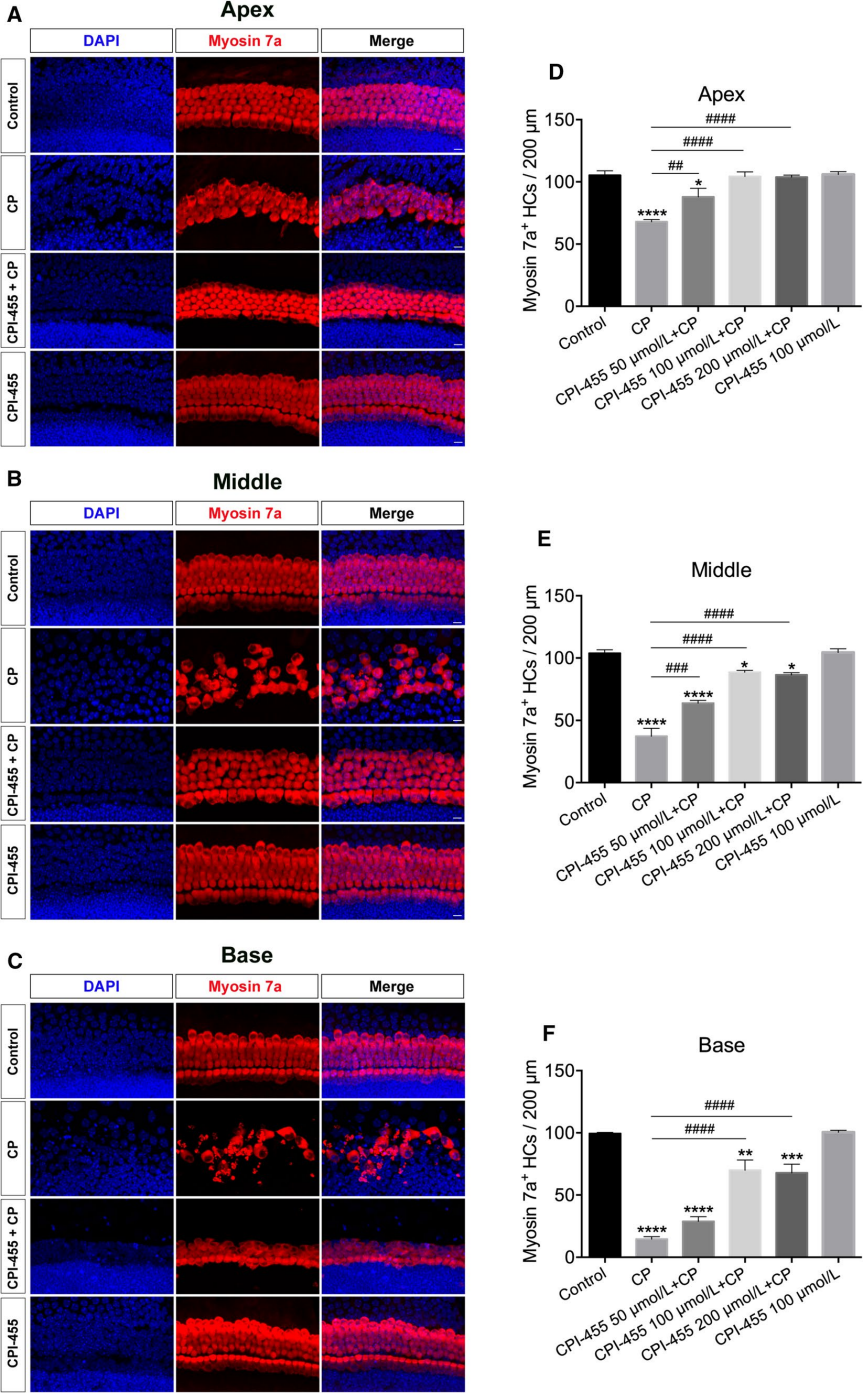
CPI-455 reduced CP-induced HC loss in ex vivo cochlear explants. **A**–**C** Cochlear explants were stained for Myosin 7a (red) and DAPI (blue). Scale bar  10 μm. **D**–**F** Graphical representation of the number of Myosin 7a–positive HCs per each treatment. The data are presented as the mean ± SEM values. *P < 0.05, **P < 0.01, ***P < 0.001, ****P < 0.0001 compared with the control; ^##^*P* < 0.01, ^###^*P* < 0.001, ^####^*P* < 0.0001 compared with CP only. The experiments were performed in triplicate. *n* = 6 cochleae for control group; *n* = 6 cochleae for CP group; *n* = 6 cochleae for 50 μmol/L CPI-455 + CP group; *n* = 6 cochleae for 100 μmol/L CPI-455 + CP group; *n* = 6 cochleae for 200 μmol/L CPI-455 + CP group; *n* = 6 cochleae for 100 μmol/L CPI-455 group

First, we investigated whether CPI-455 could protect cochlear HCs against CP damage using ex vivo whole-organ cochlear explant cultures. According to our previous study [14], cochleae from P2 wild-type (WT) mice were dissected and pretreated with different CPI-455 concentrations (50, 100, and 200 μmol/L) for 2 h, followed by treatment with or without 30 μmol/L CP for 24 h. After 2 days of recovery in medium, cultured cochlear explants were immunolabelled with the HC marker myosin 7a. As shown in Fig. 1, compared with control group, HC loss in all three (base, middle, and apex) regions of the cochlea increased significantly after CP treatment. In contrast, treatment with CPI-455 remarkably attenuated the CP-induced HC loss. Of note, both inner HCs (IHCs) and outer HCs (OHCs) were found in intact arrangements in the CPI-455 + CP group (Fig. 1A–C). Quantitative results show that, compared to the CP group, 100 μmol/L of CPI-455 showed the most significant protective effect; therefore, 100 μmol/L was used as the optimal protective concentration of CPI-455 against CP-induced HC loss (Fig. 1D–F). Furthermore, the observation of the CPI-455-only group suggested no deleterious effects on the cochlear HCs treated with 100 μmol/L of CPI-455 alone (Fig. 1A–C). These results demonstrate that CPI-455 can prevent CP-induced cochlear HC death.

**Fig. 2:**
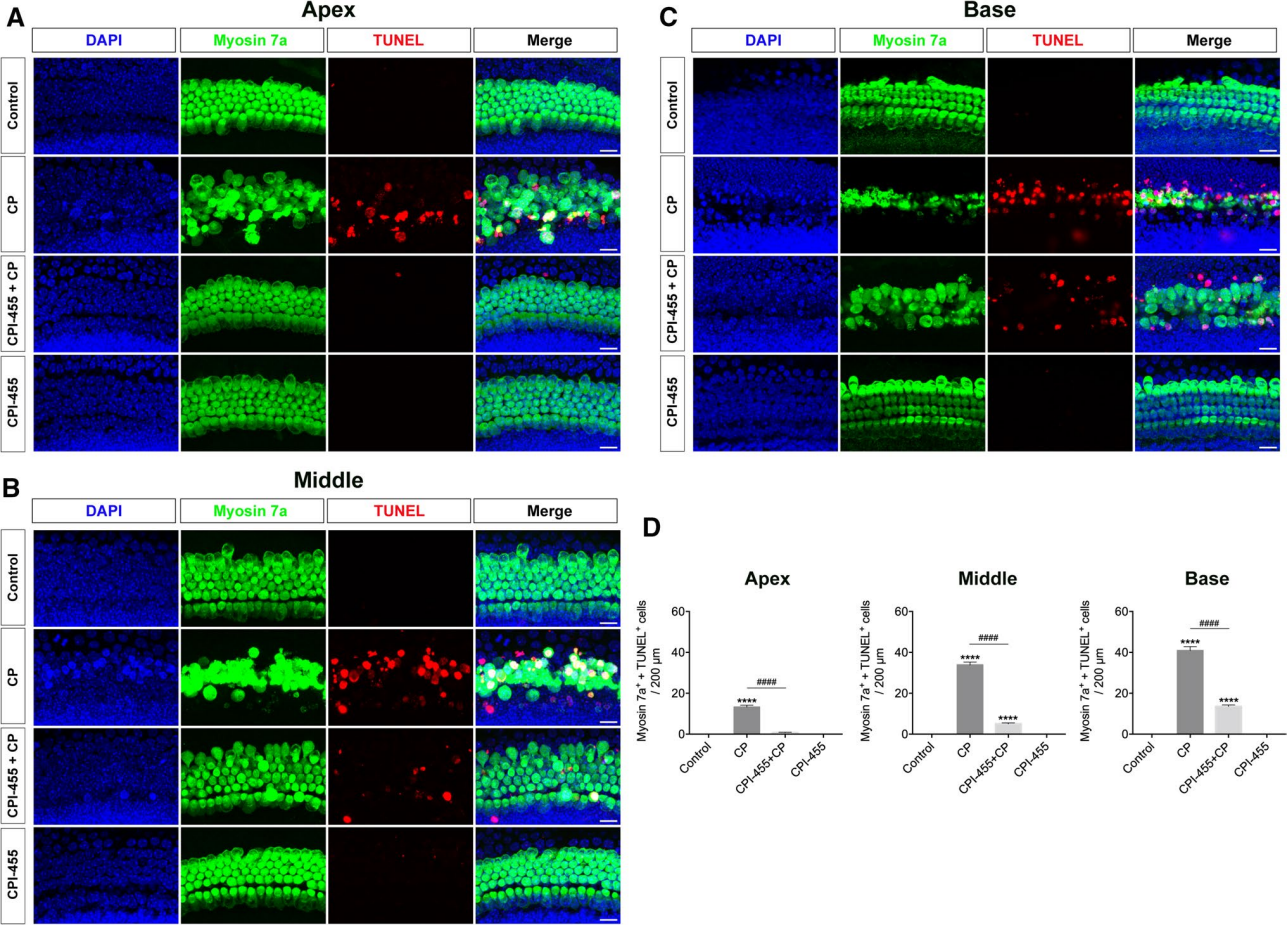
CPI-455 reduced CP-induced HC apoptosis. **A**–**C** Cochlear explants were stained with Myosin 7a (green), TUNEL (red), and DAPI (blue). Images were captured from the cochlea's apex, middle, and base turns per condition. Scale bar 10 μm. **D** Statistical plot of Myosin 7a/TUNEL-positive cells in **A**–**C**. The data are presented as the mean ± SEM. ****P < 0.0001 compared with the control and ^####^*P* < 0.0001 compared with CP only. The experiments were performed in triplicate. *n* = 8 cochleae for control group; *n* = 8 cochleae for CP group; *n* = 8 cochleae for CPI-455 + CP group; *n* = 8 cochleae for CPI-455 group

**Fig. 3:**
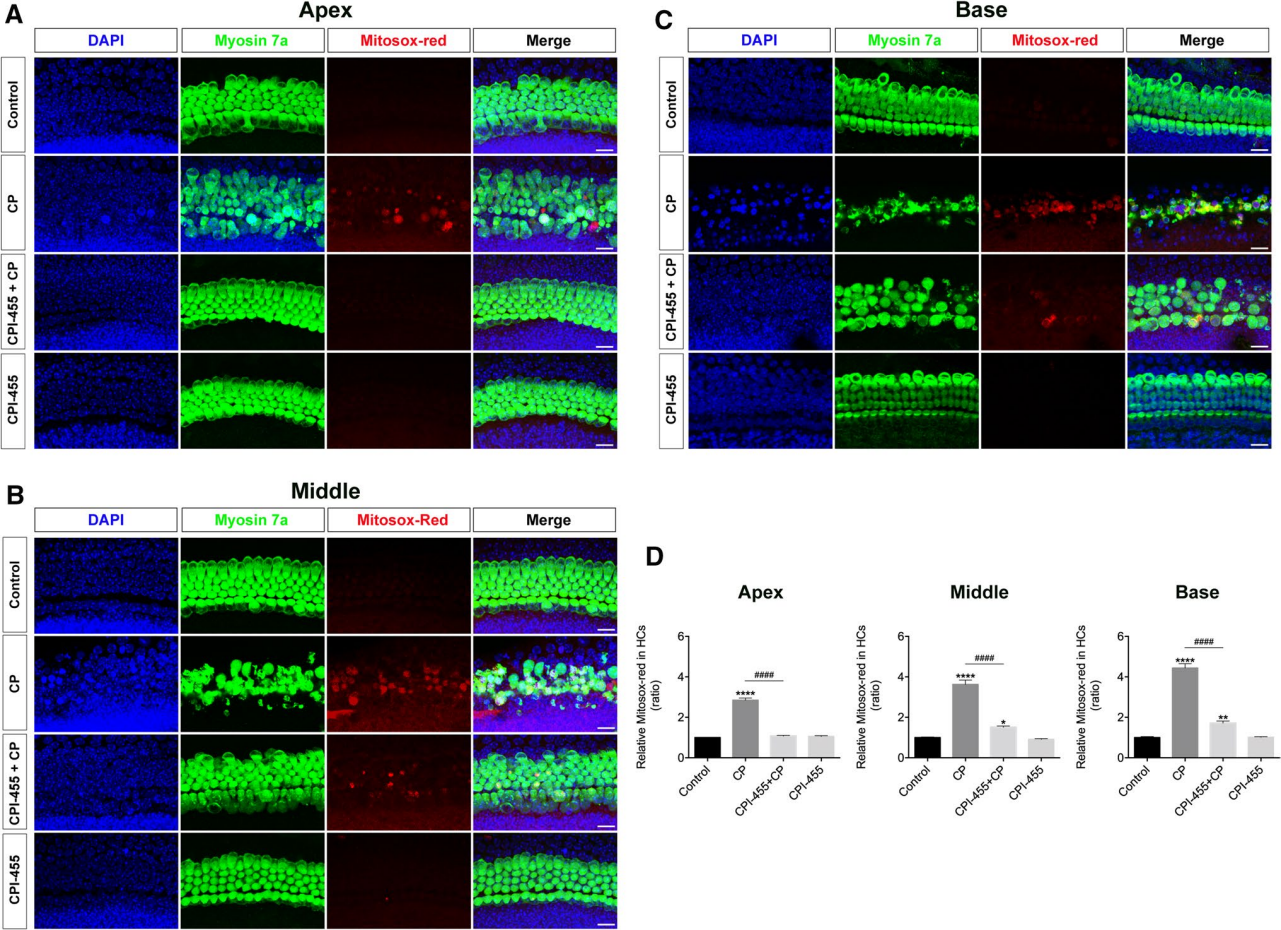
CPI-455 reduced CP-induced ROS generation in cochlear HCs. **A**–**C** Cochlear explants were stained with Myosin 7a (green), MitoSOX™ red (red), and DAPI (blue). Images were captured from the cochlea's apex, middle, and base turns per condition. Scale bar 10 μm. **D** Comparison of MitoSOX™ red signals between each group. The data are presented as the mean ± SEM. *P < 0.05, **P < 0.01, ****P < 0.0001 compared with the control; ^####^*P* < 0.0001 compared with CP only. The experiments were performed in triplicate. *n* = 9 cochleae for control group; *n* = 9 cochleae for CP group; *n* = 9 cochleae for CPI-455 + CP group; *n* = 9 cochleae for CPI-455 group

We further investigated the protective mechanisms of CPI-455 in cochlear HCs using the TUNEL assay. After exposure to CP, cochlear explants showed considerable TUNEL (red) signaling; on the contrary, the red fluorescence in CPI-455 + CP group decreased significantly compared with that of CP group (Fig. 2A–D), which indicated that CPI-455 suppressed CP-induced apoptosis in cochlear explants. Since the accumulation of reactive oxygen species (ROS) is related to CP-mediated HC apoptosis, MitoSOX™ red probe was used to detect the production of mitochondrial ROS. The results showed that cochlear explants exposed to CP displayed a significant increase in their fluorescence signal, indicating greater mitochondrial ROS generation than in the explants from the control group (Fig. 3A–D). In contrast, pretreatment of the cochlear explants with CPI-455 considerably ameliorated the CP-mediated overproduction of mitochondrial ROS (Fig. 3A–D).

### CPI-455 protects cochlear spiral ganglion neurons (SGNs) against CP ototoxicity in vitro

**Fig. 4:**
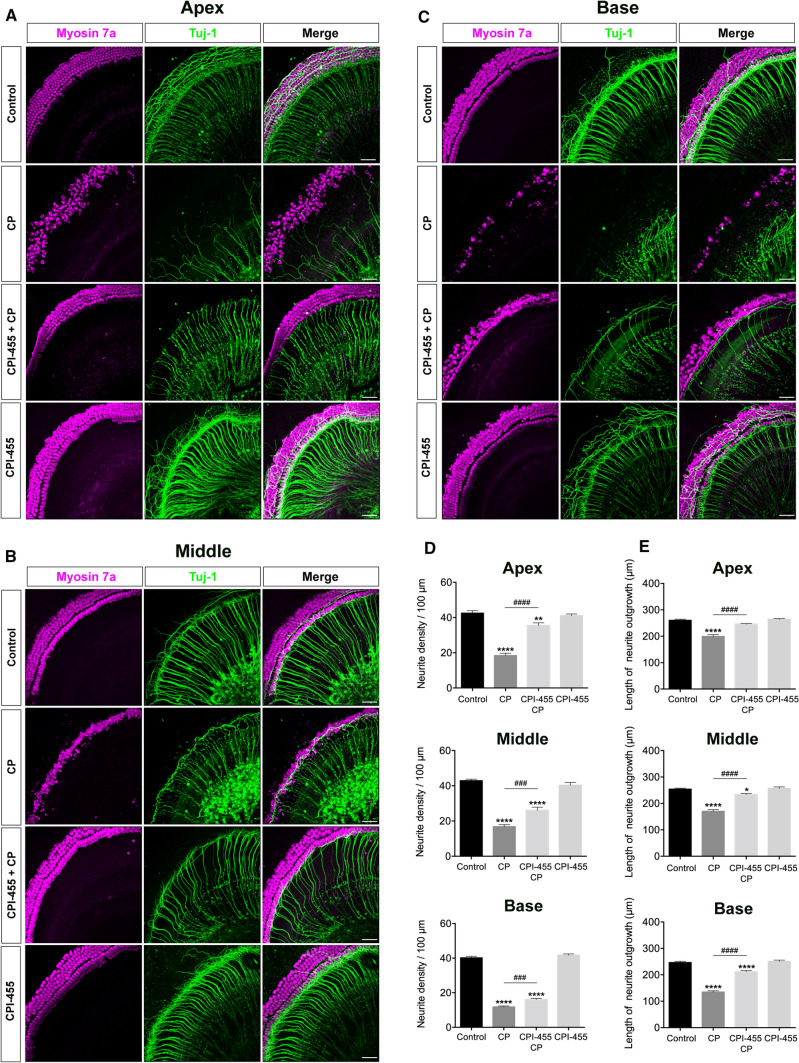
CPI-455 reduced CP-mediated SGNs loss in ex vivo cochlear explants. **A**–**C** Representative confocal photomicrographs show the spiral ganglion neurons from the apex, middle, and base turns of the cochlea treated with medium alone (control), CP-only (CP), CPI-455 + CP and CPI-455 only for 24 h and stained with Myosin 7a (magenta) and Tuj-1 (green). Scale bar 50 μm. **D**, **E** Quantification analysis of neurite densities and the length of neurite outgrowth from the apex, middle, and base turn of cochlear explants for all treatment conditions. The data are presented as the mean ± SEM. *P < 0.05, **P < 0.01, ****P < 0.0001 compared with the control; ^###^*P* < 0.001, ^####^*P* < 0.0001 compared with CP only. The experiments were performed in triplicate. *n* = 10 cochleae for control group; *n* = 10 cochleae for CP group; *n* = 10 cochleae for CPI-455 + CP group; *n* = 10 cochleae for CPI-455 group

Next, to study the effect of CPI-455 on SGNs, the cochlear SGNs of P2 mice were pretreated with a 100 μmol/L dose of CPI-455 for 2 h and then treated with or without 30 μmol/L of CP for 24 h. Figure 4A–C presents the typical confocal images of cochlear SGNs marked with Tuj-1. After CP treatment, the SGN morphological examination revealed that many nerve fibers were missing, and some blebs appeared on the remaining fibers. Compared with the untreated controls, the number of auditory nerve fibers decreased (Fig. 4D). Furthermore, the auditory nerve fiber bundles projecting from the SGNs to HCs were counted to quantify the effects of CP on the auditory nerve fibers. Neurites in the CP group were relatively shorter than those in the control group (Fig. 4E). In contrast, nerve fiber density and length were significantly increased in the CPI-455 + CP group compared with the CP-only group (Fig. 4D, E). These data suggest that CPI-455 can protect SGNs from CP-induced damage.

### CPI-455 protects HCs and ribbon synapses from CP ototoxicity in vivo

**Fig. 5:**
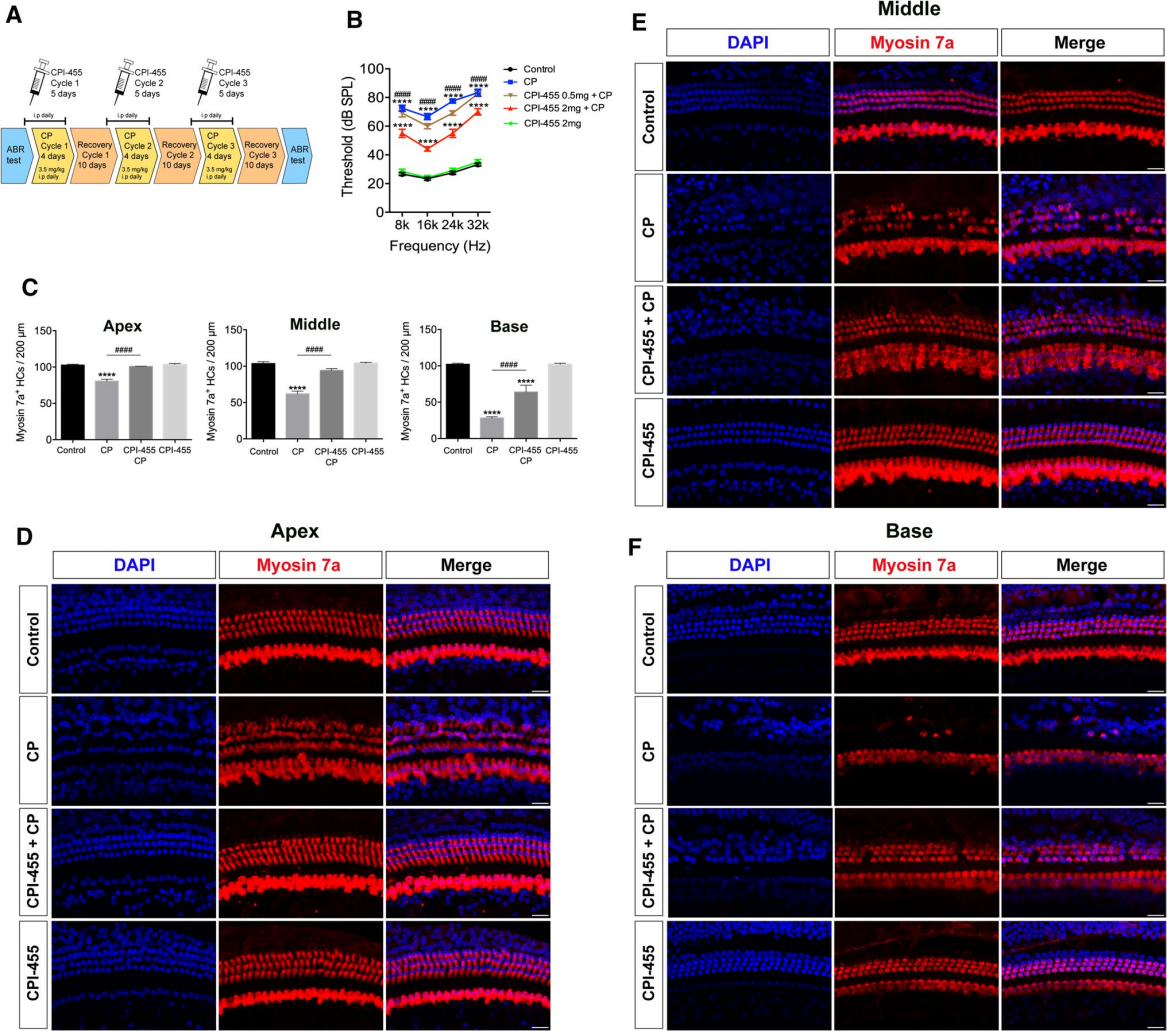
CPI-455 protected against CP-induced hearing loss in vivo. **A** Experimental workflow. **B** ABR analysis. Data are shown as mean ± SEM values. ****P < 0.0001 compared with the control; ^####^*P* < 0.0001 compared with CP only. *n* = 6 mice for each group. **C** Graphical representation of the number of Myosin 7a–positive HCs. Data are shown as mean ± SEM values. ****P < 0.0001 compared with the control; ^####^*P* < 0.0001 compared with CP only. The experiments were performed in triplicate. *n* = 10 cochleae for control group; *n* = 10 cochleae for CP group; *n* = 10 cochleae for CPI-455 + CP group; *n* = 10 cochleae for CPI-455 group. **D**–**F** Cochleae were stained with Myosin 7a (red) and DAPI (blue). Scale bar 10 µm

To investigate the effect of CPI-455 in vivo, we used a previously reported CP injury model in vivo (Fig. 5A) [23]. After CP treatment, auditory brainstem response (ABR) showed that the hearing thresholds of the CP group were higher than that of the control group at all test frequencies. Treatment with a 0.5 mg/kg dose of CPI-455 did not significantly reduce the increase in the ABR threshold caused by CP. When the dose was increased to 2 mg/kg, however, it significantly reduced the auditory threshold shift (Fig. 5B). Immunofluorescence staining with anti–myosin 7a antibody showed that the number of HCs in the apical, middle and basal cochlear regions decreased significantly after CP treatment compared with those of the control group (Fig. 5D–F). In contrast, CPI-455 treatment significantly increased the number of surviving HCs compared with that in the CP group (Fig. 5C). To better investigate the protective effect of CPI-455 on CP-induced hearing loss, we used a high-dose CP-induced ototoxicity model in C57BL/6J mice. C57BL/6J mice received a single intraperitoneal injection of 30 mg/kg of CP to induce hearing loss, and CPI-455 (10 mg/kg) was administered 2 h before CP administration. The ABR was tested 14 days after CP injection, and the results showed that treatment with CPI-455 protected against CP-induced hearing loss in the CP-induced ototoxicity model (Fig. S1 in Supporting Information). These data support the idea that CPI-455 has a significant protective effect on CP-induced hearing loss.

**Fig. 6:**
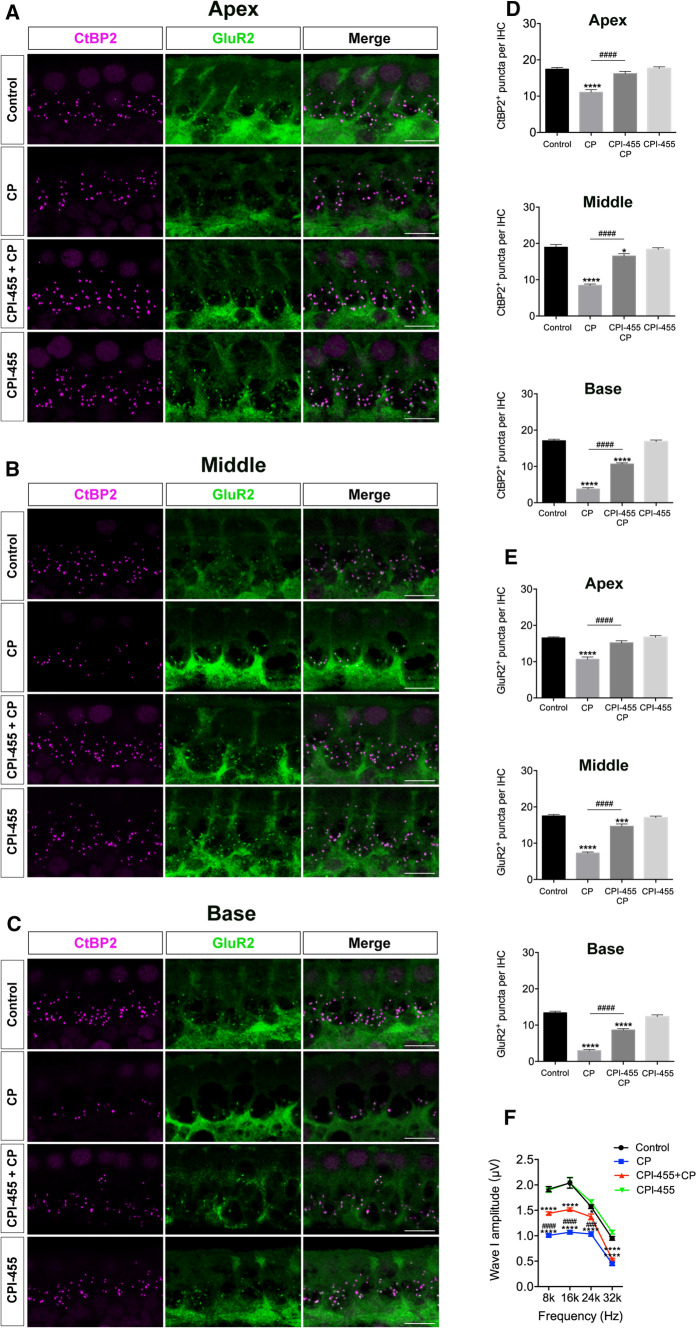
CPI-455 protected mice from CP-induced synapses loss. **A**–**C** Cochleae were stained for CtBP2 (magenta) and GluR2 (green). Scale bar 10 μm. **D**, **E** Quantification of CtBP2-immunolabeled or GluR2-immunolabeled synaptic ribbons per IHCs. The data are presented as the mean ± SEM values. *P < 0.05, ***P < 0.001, ****P < 0.0001 compared with the control; ^####^*P* < 0.0001 compared with CP only. The experiments were performed in triplicate. *n* = 6 cochleae for control group; *n* = 6 cochleae for CP group; *n* = 6 cochleae for CPI-455 + CP group; *n* = 6 cochleae for CPI-455 group. **F** ABR wave I amplitudes analysis. Data are shown as mean ± SEM values. ****P < 0.0001 compared with the control; ^####^*P* < 0.0001 compared with CP only. *n* = 6 mice for each group

Ribbon synapses help to maintain normal hearing function and are vulnerable to ototoxic stimulation [24]. To investigate the effects of CPI-455 on the synapses, we identified the ribbon synapses of IHCs by double immunofluorescent labeling of presynaptic ribbons with anti-CtBP2 antibody (magenta) and postsynaptic ribbons with anti-GluR2 antibody (green). As demonstrated in Fig. 6A–C, after CP treatment, the numbers of ribbon synapses were significantly lower than those in the control group in all three regions examined. However, CPI-455 treatment remarkably lowered the CP-mediated loss of ribbon synapses (Fig. 6D, E). As the wave I amplitude reflects characteristic features of cochlear synaptopathy, we measured the average ABR wave I amplitude in different groups. As shown in Fig. 6F, CP administration decreased wave I amplitudes compared with those of the controls, whereas CPI-455 attenuated the CP-induced decrease. Therefore, these results demonstrate that the inhibition of KDM5A prolonged HC and ribbon synapse survival and rescued CP-induced hearing loss. To investigate any potential cytotoxicity of CPI-455 on normal tissues, we collected heart, liver, spleen, and lung tissue to analyze changes. Hematoxylin and eosin (H&E)-stained sections were visualized using a light microscope. No significant differences were found in these tissues between the control and CPI-455-only groups (Fig. S2 in Supporting Information), demonstrating that CPI-455 exhibits potent anti-apoptosis activity with insignificant toxicity in vivo. Two tumor cell lines were selected to test whether CPI-455 protects tumor cells during CP treatment, including Fadu (head and neck cancer) and HGC-27 (gastric cancer) cells. The results showed that both cell lines were sensitive to CP (Fig. S3A and D in Supporting Information). When treated with CPI-455, Fadu and HGC-27 cancer cells were sensitive at high doses (Fig. S3B and E in Supporting Information). When co-treated with CP and CPI-455, further reductions in cell survival were observed in both cell lines (Fig. S3C and F in Supporting Information). The results indicated that CPI-455 had no protective effects on these tumor cell lines. Instead, a synergistic effect with CP was observed on gastric cancer and head and neck cancer cell lines.

### CPI-455 promotes the methylation level of H3K4 tri-methylation in the HCs under CP stress

**Fig. 7:**
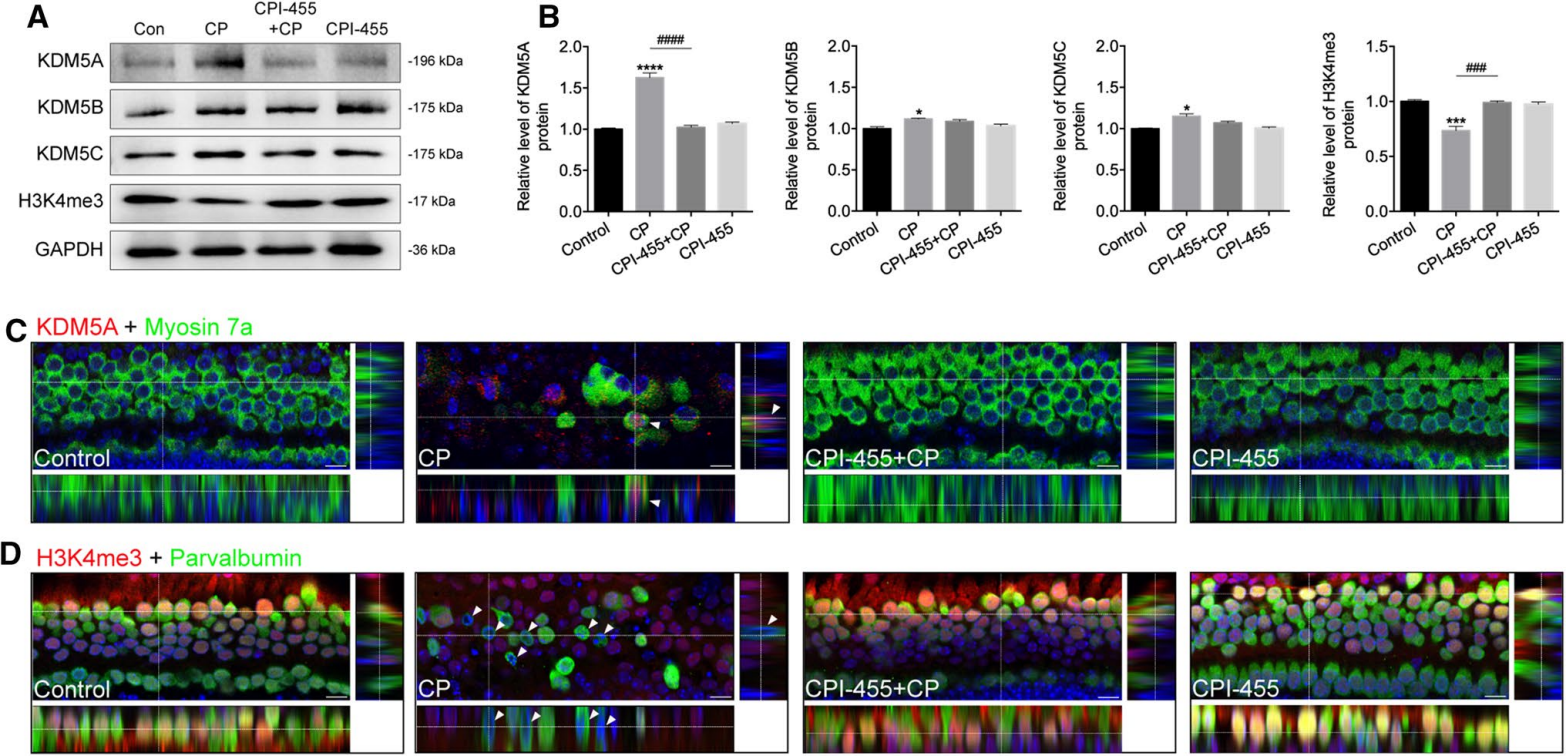
CPI-455 affected KDM5A and H3K4me3 expression in cochlear HCs. **A**, **B** The protein levels of KDM5A, KDM5B, KDM5C, and H3K4me3. GAPDH served as an internal control. Data are presented as mean ± SEM values. *P < 0.05, ***P < 0.001, ****P < 0.0001 compared with the control; ^###^*P* < 0.001, ^####^*P* < 0.0001 compared with CP only. The experiments were performed in triplicate. **C** Cochleae were stained for KDM5A (red) and Myosin 7a (green). **D** Cochleae were stained for H3K4me3 (red) and parvalbumin (green). Images were captured from the middle turns of the cochlea. Scale bars 10 μm

Since CPI-455 is well known to inhibit the activity of the histone demethylase KDM5, the protein levels of KDM5A/B/C were detected by western blot analysis. The results showed that the KDM5A signal in the CP-exposed group was significantly higher than that of KDM5B and KDM5C compared to the control group (Fig. 7A, B). Consistent with the western blotting results, immunofluorescence analysis of cochlear HCs showed that CP treatment significantly upregulated KDM5A expression in the damaged HCs (Fig. 7C). Conversely, the use of CPI-455 decreased the KDM5A level in the survival HCs under CP treatment (Fig. 7A–C). Furthermore, the overall H3K4me3 levels declined in the CP group compared with the untreated control group, while the CPI-455 treatment increased the overall level of H3K4me3 in the HCs (Fig. 7A–D). The results suggest that CPI-455 may attenuate CP-mediated HC apoptosis via KDM5A inhibition.

### Knockdown of KDM5A attenuates ROS signaling-mediated apoptosis in HEI-OC1 cells

**Fig. 8:**
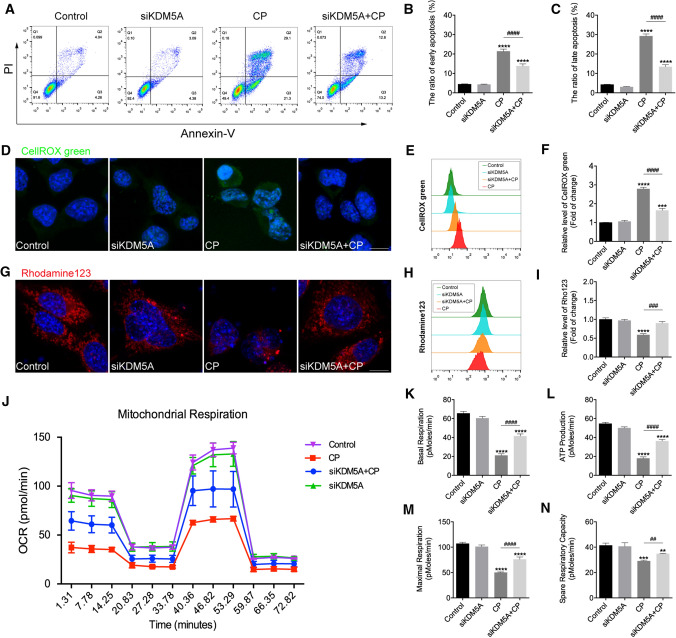
Downregulation of KDM5A reduced CP-induced apoptosis in HEI-OC1 cells. **A**–**C** Effects of KDM5A on early (Annexin V-FITC positive, PI negative) and late (Annexin V-FITC/PI double positive) cell apoptosis were (**A**) determined via flow cytometry and (**B**, **C**) quantified. The data are presented as three independent experiments' mean ± SEM values. ****P < 0.0001 compared with the control; ^####^*P* < 0.0001 compared with CP only. **D**–**F** Representative images and histogram analysis of CellROX™ green in HEI-OC1 cells. Scale bar = 10 μm. The data are presented as three independent experiments' mean ± SEM values. ***P < 0.001, ****P < 0.0001 compared with the control; ^####^*P* < 0.0001 compared with CP only. **G**–**I** Representative images and histogram analysis of Rhodamine 123 in HEI-OC1 cells. Scale bar 10 μm. The data are presented as three independent experiments' mean ± SEM values. ****P < 0.0001 compared with the control; ^###^*P* < 0.001 compared with CP only. **J**–**N** OCRs and bioenergetics parameters in HEI-OC1 cells from each group. The data are presented as mean ± SEM values. **P < 0.01, ***P < 0.001, ****P < 0.0001 compared with the control; ^##^*P* < 0.01, ^####^*P* < 0.0001 compared with CP only. The experiments were performed in triplicate

To test the above finding, we knocked down KDM5A in HEI-OC1 cells by small interfering RNA (siRNA) transfection. The transfection efficiency of siRNA-KDM5A was confirmed using qRT-PCR (Fig. S4A in Supporting Information) and western blot (Supporting Information Fig. S4B–C) assays. At 48 h after siRNA transfection, the HEI-OC1 cells were treated with 30 μmol/L of CP for 24 h. Annexin V-FITC and PI assays were carried out to detect the percentages of early apoptotic cells (Annexin V-FITC positive, PI negative) and late apoptotic cells (Annexin V-FITC/PI double positive) (Fig. 8A). We found that, after 24 h of culture without CP treatment, KDM5A inhibition itself had almost no effect on early (bottom right quadrants) and late apoptosis (top right quadrants). With CP treatment, the proportions of early and late apoptotic cells in the siRNA-KDM5A group were reduced compared with those in the CP-only group (Fig. 8B, C), suggesting that knockdown of KDM5A significantly suppressed CP-induced HEI-OC1 cell apoptosis.

Multiple studies have demonstrated that ROS is the main cause of HC death induced by ototoxic drugs [25]. To investigate whether inhibiting KDM5A protects HCs against CP-induced damage by reducing the production of ROS, we used CellROX™ green, a fluorescent probe that emits green fluorescence once oxidized by ROS, to measure the total ROS level in HEI-OC1 cells. The results showed that, after CP exposure, the levels of ROS increased significantly, while the downregulation of KDM5A could reduce the production of ROS. Flow cytometry data confirmed that HEI-OC1 cells in the CP + siRNA-KDM5A group produced significantly lower ROS than those in the CP group (Fig. 8D–F). Given that mitochondrial disorders can lead to ROS accumulation [26], we next sought to find out whether an improvement in mitochondrial function accompanied the anti-apoptosis effect of KDM5A inhibition. Changes in mitochondrial membrane potential (MMP) were investigated using Rhodamine 123 probe. As shown in Fig. 8G, MMP levels decreased significantly after 24 h of CP exposure compared to the control group, indicating that CP had an impairing effect on mitochondrial function. In contrast, knockdown of KDM5A alleviated the decline of MMP, revealing a protective role of CPI-455 in mitochondria (Fig. 8H, I). Next, we further investigated the effect of si-KDM5A on mitochondrial respiratory capacity. Oxygen consumption rates (OCRs), an index of mitochondrial respiratory capacity, were measured in HEI-OC1 cells using a Seahorse XF Cell Mito Stress Test (Fig. 8J). Following CP treatment of HEI-OC1 cells for 24 h, basal OCR, adenosine triphosphate production, and maximum and spare respiration were significantly reduced; KDM5A knockdown partially prevented these changes (Fig. 8K–N). These results confirmed that KDM5A inhibition could attenuate CP-induced mitochondrial dysfunction in HEI-OC1 cells.

### CPI-455 reduces CP-induced ototoxicity through regulating MAPK and PI3K/AKT signaling

**Fig. 9:**
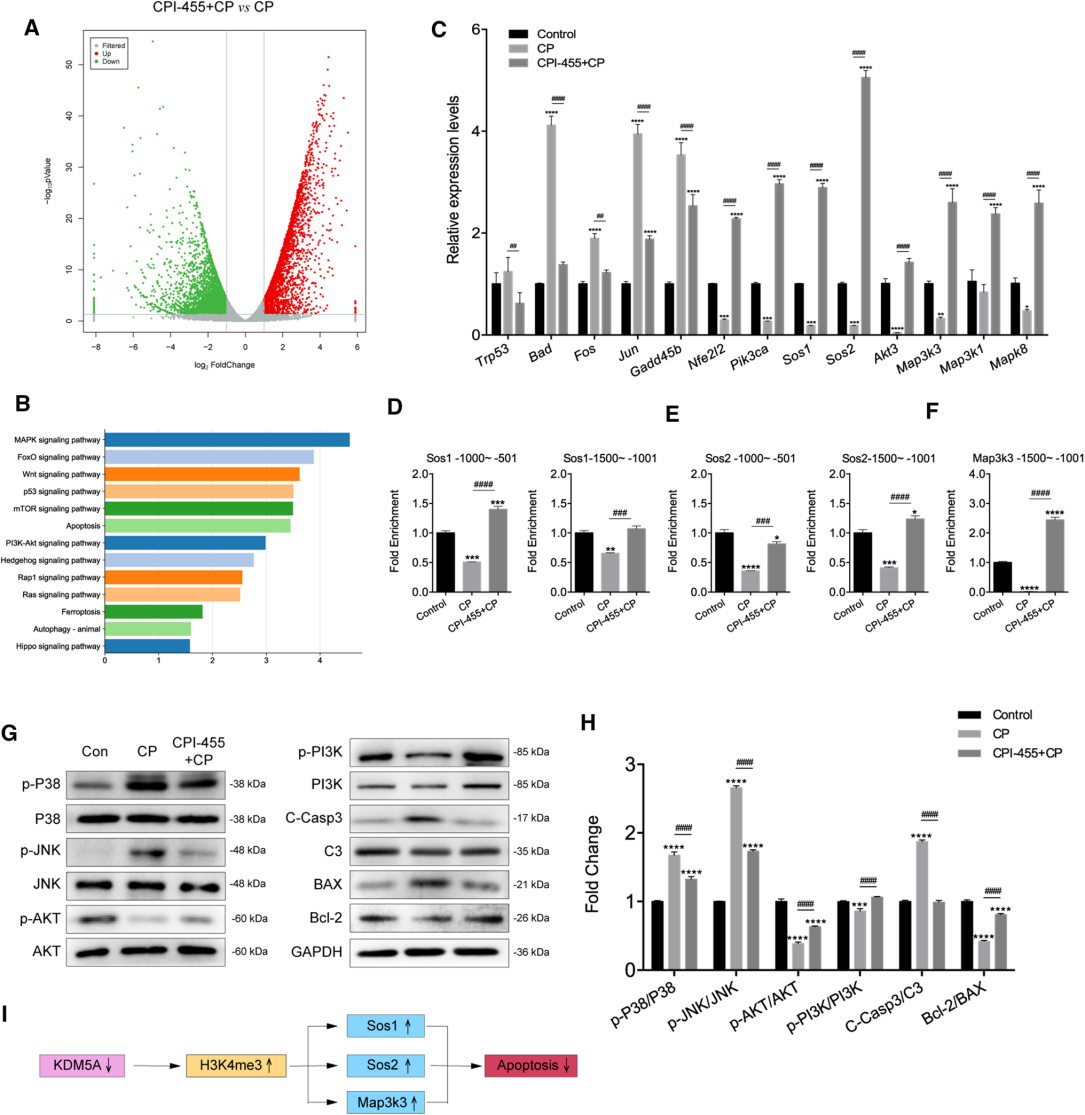
CPI-455 reduced CP-induced HC death via multiple signaling pathways. **A** Volcano plot representing gene-expression differences between CPI-455 + CP and CP. **B** KEGG analysis showed the distribution of terms exhibiting statistically significant differences. **C** The differentially expressed genes were verified in HEI-OC1 cells by qRT-PCR. Data are presented as mean ± SEM values. *P < 0.05, **P < 0.01, ***P < 0.001, ****P < 0.0001 compared with the control; ^##^*P* < 0.01, ^####^*P* < 0.0001 compared with CP only. **D**–**F** Enrichment of H3K4me3 on promoters of *Sos1*, *Sos2*, and *Map3k3* genes. Data were normalized to the IgG controls. Data are presented as mean ± SEM values. *P < 0.05, **P < 0.01, ***P < 0.001, ****P < 0.0001 compared with the control; ^###^*P* < 0.001, ^####^*P* < 0.0001 compared with CP only. **G**, **H** The protein levels of p-P38, P38, p-JNK, JNK, p-AKT, AKT, p-PI3K, PI3K, C-Casp3, C3, BAX, and Bcl-2 in HEI-OC1 cells. GAPDH serves as an internal control. Data are presented as mean ± SEM values. ***P < 0.001, ****P < 0.0001 compared with the control; ^####^*P* < 0.0001 compared with CP only. The experiments were performed in triplicate. **I** Summary of the working thesis of the study

To identify potential transcription factors regulated by CPI-455 in HEI-OC1 cells during CP-induced injury, we utilized RNA sequencing (RNA-Seq). According to the results of the RNA-Seq, 3264 were significantly downregulated, and 3318 were upregulated by CPI-455 + CP (vs. CP). Gene expression patterns were presented as a volcano map (Fig. 9A). Kyoto Encyclopedia of Genes and Genomes (KEGG) pathway enrichment analysis was conducted, and the top pathways of the enrichment analysis are shown in Fig. 9B. To verify the differentially expressed genes (DEGs), we selected some genes and detected them by qRT-PCR (Online Table 1 and Fig. 9C). Given that H3K4me3 is commonly defined as an activating histone modification and that CPI-455 produces a marked global increase in H3K4 methylation, we hypothesized that CPI-455 might promote an increase in CP-induced silent gene expression by increasing H3K4me3 levels in specific gene promoter regions. To test this, we performed CUT&Tag coupled with quantitative PCR (qPCR) assay on HEI-OC1 cells. Compared to the CP group, we observed a substantial increase in H3K4me3 within the promoters of the SOS family members *Sos1* and *Sos2* and the MAPK key regulator *Map3k3* in cells treated with both CP and CPI-455 (Fig. 9D–F). The data suggest that CPI-455 mediates the increase in H3K4me3 promoter occupancy, which may enhance the expression of a group of apoptosis-related genes.

Western blot assay was performed to verify the expression of several critical factors of the MAPK and PI3K/AKT signaling pathway and apoptosis-related proteins in each group. The expression of the pro-apoptotic proteins, including p-P38, p-JNK, BAX, and cleaved caspase-3, was significantly higher in the CP group compared to the control group (Fig. 9G). In contrast, the expression levels of anti-apoptotic proteins, including p-AKT, p-PI3K, and Bcl-2, were decreased in the CP group compared to the control group (Fig. 9G). After CPI-455 treatment, the expression of both pro-apoptotic and anti-apoptotic proteins was similar to that of the control group (Fig. 9H). Together, these results show that CPI-455 treatment might protect against CP-induced cell death by regulating MAPK and PI3K/AKT signaling.

## Discussion

Various studies show that epigenetics, such as histone modification, is closely related to inner ear development and hearing loss caused by multiple factors, so epigenetic modification may be a new therapeutic strategy [11, 12, 14]. Our previous research indicated that the expression level of LSD1 (KDM1A) in injured HCs and SGNs increases significantly after CP or neomycin damage. We also found that LSD1 by small compounds effectively inhibited apoptosis and promoted the survival of HCs and SGNs [11, 12]. These results suggested that histone methylated modification is closely involved in the auditory system. In the follow-up study on the role of crucial histone demethylases conducive to the survival of HCs, we found that KDM5A was correlated with the survival of HCs after CP-induced injury.

A growing pool of literature demonstrates that KDM5A is highly expressed in various cancer tissues, including gastric cancer, acute myeloid leukemia, and breast cancer, and proves that KDM5A is a potential molecular biomarker and novel target for cancer therapy [27–29]. In cancer treatment, KDM5A-specific inhibitors can reduce the drug resistance of cancer cells and induce tumor cell death [27, 30, 31]. KDM5A is an essential epigenetic factor that maintains neural progenitor cells in an undifferentiated state by inhibiting astrocyte differentiation [32]. Interestingly, a recent study revealed that the inhibition of KDM5A can improve the protective effect of dexmedetomidine on renal failure [33]. Therefore, KDM5A seems to regulate cell survival through different mechanisms in a context-dependent manner. However, the detailed involvement of KDM5A in ototoxic diseases has rarely been studied.

In this study, we employed a mouse model to explore the role of KDM5A in CP-induced ototoxicity. The results of western blot and immunofluorescence showed that the expression of KDM5A was upregulated in the CP injury group. However, because the proteins used for Western blot analysis were isolated from the entire cochlear epithelium, and immunofluorescence staining focused on the expression of KDM5A in the hair cell layer, the immunostaining of KDM5A could not fully reflect the changes detected by Western blot. CPI-455 was designed as a cell-permeable prodrug, which can change the level of H3K4me3, thus laying the foundation for the further development of therapeutic drugs against KDM5 potential [34]. By employing mouse in vitro and in vivo CP injury models, we found that the inhibition of KDM5A histone lysine demethylase with CPI-455 protected against CP-induced hearing loss. C57BL/6J mice have been widely used as an optimal mouse model for the study of age-related hearing loss. These mice maintain normal hearing for 13 weeks, then display the classic pattern of age-related hearing loss, i.e., high-frequency hearing loss, beginning around 13–26 weeks of age, and the loss progresses toward the low frequency [35–39]. Hearing loss was reported at all frequencies up to 65 weeks, with no measurable hearing response at 78 weeks [40]. Previous studies have shown that loss of ribbon synapses is the initial pathological event in the early aging stage [41, 42]. Xiong et al. showed that younger C57BL/6J mice (< 4 months) had an average number of HCs, SGNs, and synapses; however, for mice aged 4 months, the number of cochlear ribbon synapses was reduced in the high-frequency cochlear regions, and significant elevations in the hearing threshold initially appeared at a high frequency at 4 months and across frequencies at 6 months, which suggests that the lifespan of 4 months was the early stage of aging [41]. In our in vivo experiment, to be consistent with the in vitro data, C57BL/6J mice were used and treated with CP using a three-cycle regimen. At the time of analysis, the mice were 14–15 weeks (< 3.5 months) old. Although this mouse model was < 4 months old, with normal hearing and an average number of HCs and synapses, to illustrate better the protective effect of CPI-455 on CP-induced hearing loss and exclude the influence of age-related hearing loss factors, we used a C57BL/6J mouse ototoxicity model with a high dose of CP. Our result showed that the administration of CP at 30 mg/kg caused significant elevations in the hearing threshold, and CPI-455 treatment protected against CP-induced hearing loss in this mouse model. Taken together, these data supported the idea that CPI-455 could be used as a candidate therapeutic agent for protection against CP-induced ototoxicity.

Oxidative damage would impair mitochondrial function and cause cell death. In this study, we showed that knockdown of KDM5A reduced CP-induced production of ROS and disruption of MMP in HCs, suggesting that KDM5A inhibition might achieve its otoprotective effect by inhibiting the generation of ROS and maintaining the integrity of mitochondria. As an indicator of mitochondrial function, the dysfunction of MMP might lead to a proton gradient within the inner mitochondrial membrane. In a healthy mitochondrion, the net negative charge is majorly generated by cytochrome *c*. However, when the mitochondrial function is impaired by ototoxic disease, the consequences are mitochondrial membrane permeabilization and cytochrome* c* release into the cytosol, causing an immediate dissipation of △ψm. It is speculated that the destruction of MMP may inhibit the respiratory capacity of mitochondria. Previous studies have shown that the loss of KDM5A could increase mitochondrial respiration, thus restoring the differentiation of mouse embryonic fibroblasts, suggesting that KDM5A may be an epigenetic regulator that controls metabolic processes and synchronizes energy homeostasis with differentiation signals [18]. Our study examined whether knockdown of KDM5A could improve the impaired OXPHOS activity in HEI-OC1 under CP treatment. As shown in Fig. 8J, our results indicate that KDM5A knockdown significantly increases mitochondrial OCRs, suggesting that KDM5A inhibition may be a potential approach to prevent ototoxicity.

Although KDM5A inhibition restored mitochondrial function by reducing mitochondrial ROS generation and recovering CP-treated cells' respiratory ability, the potential mechanisms were not fully elucidated. To determine the potential target of KDM5A inhibition for protection, we performed RNA-Seq analysis in HEI-OC1 cells. Of the crucial enriched pathways, PI3K/AKT and Ras/MAPK, the ROS-sensitive regulators, are two significant mediators of CPI-455 for HC protection. The PI3K/AKT signal pathway has been shown to regulate diverse cellular functions in different systems, including apoptosis, proliferation, and metabolism [43]. Many studies have demonstrated that the PI3K/AKT pathway is a crucial signaling pathway in regulating HC development, apoptosis, and the proliferation of otic progenitors [44]. The PI3K/AKT pathway participates in the survival and protection of auditory HCs by inhibiting pro-apoptotic signals and mitochondria-produced ROS [45, 46]. Zhang et al*.* demonstrated that superoxide dismutase 2 overexpression counteracted the noise and kanamycin-induced hearing loss by inhibiting MAPK and restoring the expression of PI3K [47].

Ras protein is a crucial signal regulator regulating the proliferation, differentiation, migration, and survival of different types of cells [48]. The Son of the sevenless (SOS) family of Ras-GEF contains two widely expressed members (SOS1 and SOS2), which play a role in various signals in mammalian cells and promote Ras activation [49]. A prior analysis of conditional knockout mice showed that *Sos1* and *Sos2* play an essential role in homeostasis and overall organism survival [50]. *Sos1* depletion leads to specific phenotypic defects in MEFs, including alterations of mitochondrial morphology, mitochondrial dysfunctions, and metabolic defects [50–53]. Theard et al*.* [54] indicated that combined SOS1 and estimated glomerular filtration rate inhibition is an important target in estimated glomerular filtration rate-mutated lung adenocarcinoma. Recent studies showed that SOS activates membrane-tethered Ras and triggers MAPK cascade [55], while inhibition of the Grb2–Sos interaction downregulates Raf/MEK/ERK signaling [56]. The basic composition in MAPK cascades includes MAP3Ks, MAP2Ks, and MAPKs, which are highly conserved from yeast to humans. These three kinases are activated in turn, together regulating various physiological processes, including control of cellular proliferation, growth, differentiation, and inflammation [57]. MAP3K3, also known as MEKK3, belongs to the MAP3K superfamily. MAP3K3 is the key kinase of the MAPK signaling pathway and plays a crucial role in cell proliferation, apoptosis, and inflammatory and immune responses [58]. Previous studies showed that MAP3K3 plays a role in promoting cell survival, and MAP3K3 could promote mitochondrial degradation through the MAP3K3/MEK5/ERK5 pathway, thus increasing the content of mitochondria [59], while overexpression of *Map3k3* offers resistance to apoptosis via activating the nuclear factor κB signal pathway [60]. More recently, Yuan et al. determined that the *Map3k2/3* deficiency led to increased total ROS from neutrophils upon stimulation by fMLP, MIP2, or PMA, indicating the importance of kinase activity in the regulation of ROS release [61].

Our current H3K4me3-CUT&Tag-qPCR data revealed lower enrichment of H3K4me3 at the promoters of *Sos1* and *Sos2* and in *Map3k3* genes in CP-treated cells compared to control cells, suggesting that *Sos1*, *Sos2*, and *Map3k3* are epigenetic targets of KDM5A. However, the expression of these genes increased following the addition of CPI-455 to CP-treated cells, which is related to the increased occupation of H3K4me3 in the promoter region of these genes. Our study revealed that H3K4me3-dependent epigenetic activation of MAPK target genes was associated with HC survival, but the specific molecular mechanism in HC biology need further investigation. We still do not understand how CPI-455-mediated H3K4me3 is fully recognized, affecting other epigenetic modifications to regulate gene expression and inhibit HC apoptosis. Detailed CUT&Tag-seq analysis of CPI-455-mediated H3K4me3 and other histone marks would facilitate understanding their roles in transcriptional regulation. The mechanisms through which MAPK and PI3K/AKT modulate HC senescence have not been fully clarified. Epistasis between MAPK and PI3K/AKT can be further analyzed by in vivo pharmacogenetic intervention.

Despite the encouraging results reported here, this study still has some limitations. For example, KDM5A histone lysine demethylase inhibitor CPI-455 was reported to induce apoptosis and inhibit the proliferation of many tumor cells, such as cervical [62] and prostate cancer [63]. Our study observed synergistic effects between CPI-455 and CP in tumor suppression. Therefore, it is unlikely to interfere with the therapeutic effect of CP during tumor chemotherapy. Various KDM5A target genes were identified through a genome-wide study, which is necessary for mitochondrial function or DNA metabolism [21]. In tumor cells, by removing H3K4me3, KDM5A inhibits multiple tumor suppressor genes involved in the cell cycle [19], invasion [64], epithelial–mesenchymal transition [65], and other processes that contribute to tumor progression [66, 67]. CPI-455 treatment elevated global levels of H3K4me3 and reduced the number of drug-resistant persistent cancer cells in multiple tumor cell line models receiving antitumor drugs [34]. Based on the analysis and preliminary validation of mitochondrial function and multi-omics data, we concluded that CPI-455 is critical to ROS reduction and could improve mitochondrial function by activating Ras/MAPK and PI3K/AKT signaling, which is a fundamental mechanism for protecting HC from oxidative damage. In other words, KDM5A plays an important role in both normal tissue protection and tumor suppression and is, therefore, a promising target for CP-induced ototoxicity. Future work to gain more insights into the function of CPI-455 in chemosensitivity induced by CP is warranted, and the balance of KDM5A levels in ototoxic diseases also needs to be considered when developing KDM5A inhibition-based therapies. In addition, we confirmed the protective role of KDM5A inhibition in CP-induced SGN injury, although this may not fully reflect the situation in adult mice in vivo*.* In future work, we will evaluate the protective effect of CPI-455 on SGNs using CAP recording, which reflects the total cochlear nerve output from IHC to the auditory nerve.

Adeno-associated virus (AAV) is an effective vector for treating hearing loss. Many studies aim to restore hearing in mouse hearing loss models using AAV vectors. For example, Gu et al. demonstrated the protective effect of the AAV–CRISPR/Cas9–*Htra2* system against neomycin-induced ototoxicity in vitro [68]. However, in vivo, no significant protective effect was observed in cochlear basal OHCs due to the low transduction efficiency of Anc80L65 in OHCs [69, 70]. Iizuka et al*.* attempted to restore auditory function in *Gjb2* conditional knockout mice (P42) by delivering AAV5-*Gjb2* [71], and Pan et al*.* delivered AAV2/Anc80L65-*Ush1c* in Usher syndrome type 1c knock-in mice (P10–12) to restore the hearing [72]. However, both experiments failed to rescue the auditory function of the treated mice, possibly due to low transduction efficiency or the closed therapeutic window. To further realize the clinical transformation of this treatment strategy based on AAV–CRISPR/cas9, in-depth research is needed to improve transfection efficiency, select the appropriate therapeutic window and surgical method, and evaluate the longer term safety profile. Our findings showed that inhibition of KDM5A led to an upregulated H3K4me3 level and the expression level of MAPK and PI3K/AKT target genes and promoted HC survival and hearing function after CP exposure both in vitro and in vivo. In future work, we will select the appropriate AAV–CRISPR/Cas9 system and optimal surgical procedures to verify the role of KDM5A in hearing protection.

## Conclusion

The present study showed that KDM5A participates in CP-induced hearing loss. KDM5A inhibition could protect against CP-induced ototoxicity, functioning via downregulation of oxidative stress, ROS production, and restoration of mitochondrial bioenergetic dysfunction. In addition, KDM5A inhibition reduces apoptosis by regulating a set of survival signaling pathways. Our findings have broad significance and call for the transformation of KDM5A as a potential therapeutic target for the treatment of hearing loss in the future.

## Materials and methods

### Compounds

CPI-455 (S8287; Selleckchem, Houston, TX, USA) was dissolved as 10 mmol/L of stock solution with DMSO (D5879; Sigma-Aldrich, St. Louis, MO, USA). CP was purchased from Sigma-Aldrich (479,306; Sigma-Aldrich) and was initially dissolved in PBS at a stock concentration of 3 mmol/L and further diluted in the medium used for cell and explant culture analysis.

### Cell cultures

HEI-OC1 cells were cultured in high-glucose DMEM (11965-092; Gibco, Thermo Fisher Scientific, Inc., Waltham, MA, USA) supplemented with 10% FBS (10099-141; Gibco) at 33 °C under a 10% CO_2_ condition [73–77]. FaDu and HGC-27 cell lines, preserved in our laboratory, were used before the 15th passage, and all experiments were performed in triplicate. Both cells were cultured in RPMI-1640 medium (C11875500BT; Gibco) containing 10% FBS and penicillin–streptomycin (15140-122; Gibco) under a 5% CO_2_ condition. The cells were trypsinized with 0.25% trypsin/EDTA (25200056; Gibco) at 80% confluence.

### Postnatal cochlear and SGN explants cultures and drug treatments

Animal experiments and research protocols were in accordance with the Fudan University Institutional Animal Care and Use Committee. Cochlea or SGNs explants from C57BL/6 mice at postnatal day 2 were dissected in PBS and attached to cell-Tak-coated coverslips. Then, explants were cultured in DMEM/F12 (C11330500BT; Gibco), containing 1% N2 (100 ×, 17502-048; Gibco) supplement, 2% B27 (50 ×, 17504-044; Gibco) supplement, and ampicillin (P0781; Sigma-Aldrich) at 37 °C and 5% CO_2_.

### In vivo study

Adult 8-week-old C57BL/6 mice were randomly divided into the following groups: control, CP, CPI-455, and CPI-455 + CP groups. 1 mg/mL CP stock solution was prepared in sterile saline (0.9% NaCl). The mice in the CP group received three rounds of once-daily intraperitoneal injections of CP at a dose of 3.5 mg/kg for 4 days, followed by a 10-day recovery period (a total of 42 days), as previously described [23, 78]. Mice in the CPI-455 + CP group received intraperitoneal injections of CPI-455 (0.5 mg/kg or 2 mg/kg) starting 1 day before the injection of CP, and each cycle lasted for 5 days. Mice in the control and CPI-455 groups were injected with the same volume of saline or CPI-455, respectively.

For the high-dose CP-induced ototoxicity experiment, C57BL/6 mice were injected intraperitoneally with 1 mL of warm saline. After 24 h, CPI-455 was injected intraperitoneally 2 h in advance in mice of the CPI-455 + CP group and CPI-455 group; then, a single dose of CP (30 mg/kg) was injected intraperitoneally in mice of the CPI-455 + CP group and CP group. The ABR threshold was measured 14 days after administration.

### ABR

ABR recording was implemented with the TDT system (Tucker Davis Technologies, Alachua, FL, USA). Mice were commonly anesthetized using a ketamine (100 mg/kg)–xylazine (10 mg/kg) cocktail before measuring ABR. The recording, reference, and ground electrodes were inserted at the midpoint of the line joining the anterior edge of the auricle on each side, behind the tested ear, and behind the contralateral ear of the mouse. The SigGenRZ software (Tucker Davis Technologies) performed the ABR tests at 8-, 16-, 24-, and 32-kHz frequencies. The tone bursts were selected to evoke ABR, and the sound intensity was attenuated gradually from 90 to 20 dB at intervals of 5 dB. The response for each frequency level was analyzed, digitized, and averaged using the BioSigRZ software (1024 samples/level). The amplitude of ABR wave I was determined by analyzing ABR waveforms.

### ROS detection

ROS production was detected using CellROX™ green (C10444; Invitrogen, Carlsbad, CA, USA) and MitoSOX™ red (M36008; Invitrogen) according to the method described previously [79, 80]. For analyzing ROS levels in HEI-OC1 cells, cells were incubated with 5 μmol/L of CellROX™ green (excitation/emission maxima of 488/520 nm) working solution at 37 °C for 30 min in the dark. Then, HEI-OC1 cells were stained with 10 µg/mL Hoechst 33,258 (excitation/emission maxima of 350/461 nm) (861,405; Sigma–Aldrich) for 5 min, and the images were observed and captured with a confocal fluorescence microscope (Leica TCS SP8; Leica Microsystems GmbH, Wetzlar, Germany). Fluorescent signal intensity was taken with flow cytometry. Flow cytometric analysis used a Beckman Coulter MoFlo XDP (Miami, FL, USA) with FlowJo software (Version 10; TreeStar, Ashland, OR). CellROX™ fluorescence intensity was detected at an excitation wavelength of 488 nm and an emission wavelength of 520 nm (FL-1) by flow cytometry with gating at 20,000 cells/sample and the mean fluorescence intensity used to represent the amount of ROS. All experiments were repeated three times.

Regarding ROS assessment in the cochlea, cochlear explants from different treatments were incubated with 5 μmol/L of MitoSOX™ red (excitation/emission maxima of 510/580 nm) at 37 °C for 30 min in the dark. Fluorescent signal intensity was taken with fluorescence microscopy. Briefly, the representative images of the cochleae in each turn were randomly captured with a confocal fluorescence microscope under the same settings. MitoSOX™ red immunofluorescence was quantified by ImageJ software (National Institutes of Health, Bethesda, MD, USA) from raw confocal images that were taken under the same conditions at 63 × magnification lens with a 0.75 × digital zoom and with the same laser gain and PMT gain parameters. Cochlear explants were stained with a rabbit polyclonal antibody to Myosin 7a (1:500, 25–6790; Proteus Biosciences, Ramona, CA, USA) to determine comparable portions of the HCs in confocal images. Grayscale values were determined in the HCs to quantify changes in fluorescence intensity. The MitoSOX™ red signal was measured in the apical, middle, and basal turns of cochlear explant. The intensity of the background was subtracted and the mean intensity was then calculated. For each replicate, the relative fluorescence intensity values were determined by ratio normalization with the control group.

### MMP measurement

MMP level was measured with Rhodamine 123 (C0062; LifeSct LLC, Rockville, USA) staining [79]. HEI-OC1 cells were incubated with 1 μmol/L of Rhodamine 123 at 37 °C for 30 min. Cells were then visualized under the same settings using a confocal fluorescence microscope.

For analyzing MMP levels in HEI-OC1 cells, cells were treated with trypsin, centrifuged at 1000 rpm for 5 min, and then rinsed twice with PBS. Next, HEI-OC1 cells were incubated in the dark with 1 μmol/L of Rhodamine 123 for 30 min at 37 °C, followed by flow cytometric analysis using the FACScan flow cytometer (Beckman Coulter) for a cell count of 20,000, and the data were analyzed using FlowJo software. Rhodamine 123 fluorescence intensity was detected at an excitation wavelength of 488 nm and an emission wavelength of 534 nm and the mean fluorescence intensity used to represent the levels of MMP. The experiments were repeated three times.

### Apoptosis detection

For the TUNEL (terminal deoxynucleotidyl transferase-mediated dUTP nick-end labeling) (11684795910; Roche Holding, Basel, Switzerland) test, cochlear explants were washed with PBS for 15 min and then treated with the reaction mixture at 37 °C for 30 min. Myosin 7a and DAPI were used for HCs and nuclei counterstain, respectively. Representative images of each turn of the cochleae were captured randomly with a confocal fluorescence microscope under the same settings. The number of Myosin 7a/TUNEL-positive cells were counted in the images, and the results were averaged for three images per turn (apex, middle, and base) and expressed as data per 200 µm.

For the Annexin V conjugated to FITC (556547; BD Biosciences, San Jose, CA, USA) assay, HEI-OC1 cells were harvested and washed in PBS, then 2 × 10^5^ cells were resuspended in a solution containing Annexin V (5 μL) and PI (5 μL) and incubated for 20 min. After incubation, 300 μL of binding buffer was added and the cells were analyzed by flow cytometry. All experiments were repeated three times. Cells with positive Annexin V‑FITC staining and negative PI staining were considered to be undergoing early apoptosis, and cells with positive Annexin V‑FITC and PI staining were considered to be undergoing late apoptosis.

### Cell viability detection

CCK-8 kit (CK04; Dojindo Laboratories) was used to measure the viability of the cells. Briefly, HEI-OC1 cells were seeded in 96-well plates. After drug treatment, 10 μL of Cell Counting Kit-8 solution and 90 μL of culture medium were mixed, added to each well, and incubated for 3 h. Absorbance was measured at 450 nm using a microplate reader (Bio-Rad Laboratories, Hercules, CA, USA).

### Seahorse assay

For evaluating the energy metabolism, HEI-OC1 cells were seeded in 180 μL of DMEM containing 10% FBS on Seahorse XF96-cell culture microplates (Seahorse Bioscience, Billerica, MA, USA) at the density of 1.5 × 10^4^ cells/well. Cells were washed with ~ 180 μL of XF assay medium before the assay, then cultured at 37 °C for 60 min. OCR was detected by the Seahorse XF96 instrument (Seahorse Bioscience) following the injections of mixtures in the Seahorse XF Cell Mito Stress Kit (Seahorse Bioscience). The following mixtures were added in proper order: first, oligomycin (complex V inhibitor; 25 µL in port A) at a concentration of 2 μmol/L; second, carbonyl cyanide 4-(trifluoromethoxy) phenylhydrazone (FCCP; 25 µL in port B) at the concentration of 0.5 mmol/L; and, finally, rotenone/antimycin A (25 µL) (inhibitors of complex I and III; in port C) at the concentration of 0.1 μmol/L/1 μmol/L. The XF Cell Mito Stress Test Report Generator software (version 2.6.0.31) was used to calculate and integrate the experiment data, and the results were obtained after triplicate repetition. OCR normalization was defined as total protein/well.

### KDM5A-siRNA transfection

HEI-OC1 cells (1 × 10^3^) were seeded in six-well plate and transfected with KDM5A-siRNA at a 50 nmol/L concentration with Lipo 3000 Transfection Reagent (L3000001; Invitrogen) for 24 h. The transfection efficiency of knockdown was detected by qRT-PCR. The following KDM5A-siRNA was used: (1) KDM5A siRNA-01, sense: 5′-GCACAAUCCUAUGACACUUGG-3′, antisense: 5′-CCAAGUGUCAUAGGAUUGUGC-3′. (2) KDM5A siRNA-02, sense: 5′-GCAAAUGAGACAACGGAAA-3′, antisense: 5′-UUUCCGUUGUCUCAUUUGC-3′.

### Immunofluorescence

Samples fixed with 4% PFA were permeabilized with 1% PBST for 30 min. Afterward, we blocked with 10% donkey serum in PBST for 1 h, followed by incubation with the following primary antibodies: Myosin 7a rabbit polyclonal antibody (1:500, 25–6790; Proteus Biosciences, Ramona, CA, USA), Tuj-1 mouse (IgG2a) monoclonal antibody (1:1000, 801202; Biolegend, USA), parvalbumin mouse monoclonal antibody (1:1000, ab277625; Abcam, Cambridge, MA, USA), CtBP2 mouse (IgG1) monoclonal antibody (1:1000, 612044; BD Transduction Laboratories, BD Biosciences), GluR2 mouse (IgG2a) monoclonal antibody (1:1000, MAB397; Millipore Corp., Billerica, MA, USA), H3K4me3 rabbit monoclonal antibody (1:500, 9751; Cell Signaling Technology, CST Danvers, MA, USA), and KDM5A rabbit monoclonal antibody (1:500, 3876; CST). Samples were then washed with PBS, followed by incubation with Alexa Fluor 488 donkey anti-rabbit/mouse or cy3-conjugated secondary antibody (1:1000, Invitrogen) along with DAPI (1:2000, D9542; Sigma-Aldrich) for 2 h. For image acquisition, all images were scanned with a laser scanning confocal fluorescence microscope (SP8; Leica) with a 40 × oil immersion objective lens as seen in Fig. 4A–C, or 63 × oil immersion objective lens as seen in Figs. 1A–C, 2A–C, 3A–C, 5D–F, 6A–C, 7C–D, 8D, G, and S1D–F under identical z-stack conditions (in 1 μm Z-steps) and the same laser intensity, gains, and PMT gains parameter settings.

### H&E staining

After mice were sacrificed, their hearts, livers, spleens, and lungs were harvested for H&E staining. The sections were deparaffinized twice with xylene (for 20 min each), then rehydrated twice with 100% alcohol (for 5 min each), then rehydrated with 75% alcohol (for 5 min each) and rinsed with water. The sections were stained with Harris hematoxylin solution, counterstained with eosin solution, then dehydrated three times in 100% alcohol (5 min each), and washed twice with xylene (5 min each). Finally, these sections were observed under a light microscope.

### Western blot analysis

Protein lysate from cells and cochlear sensory epithelia for each group was prepared in RIPA buffer (P0013B; Beyotime Biotech) with the addition of protease inhibitor (04693132001; Sigma-Aldrich), followed by placement on ice for 30 min, being shaken every 10 min. After the centrifugation at 12,000 rpm at 4 °C for 10 min, the supernatants were denatured and loaded on 12% SDS-PAGE gel. Proteins were then transferred in an ice bath to polyvinylidene difluoride membrane (Immobilon-P, IPVH00010; Millipore, Schaffhausen, Switzerland). After blocking with TBST buffer containing 5% milk for 1 h, the membrane was incubated with primary antibodies overnight at 4 °C, followed by secondary antibody. The antibodies were as follows: cleaved caspase-3 rabbit polyclonal antibody (1:500, 9661; CST), caspase-3 rabbit polyclonal antibody (1:500, 9662; CST), Bax rabbit monoclonal antibody (1:500, 14,796; CST), Bcl-2 rabbit monoclonal antibody (1:500, 3498; CST), phospho-Akt (Ser473) rabbit monoclonal antibody (1:500, 4060; CST), Akt rabbit monoclonal antibody (1:500, 4691; CST), phospho-PI3K rabbit monoclonal antibody (1:500, 17,366; CST), PI3K rabbit monoclonal antibody (1:500, 4257; CST), phospho-p38 MAPK (Thr180/Tyr182) rabbit monoclonal antibody (1:500, 4511; CST), p38 MAPK rabbit polyclonal antibody (1:500, 9212; CST), phospho-JNK rabbit monoclonal antibody (1:1,000, ab124956; Abcam), JNK rabbit monoclonal antibody (1:1,000, ab179461; Abcam), KDM5A rabbit monoclonal antibody (1:500, 3876; CST), KDM5B rabbit polyclonal antibody (1:500, 3273; CST), KDM5C rabbit monoclonal antibody (1:500, 5361; CST), H3K4me3 rabbit monoclonal antibody (1:500, 9751; CST), horseradish peroxidase-linked donkey anti-rabbit IgG polyclonal antibody (1:3,000, 406,401; Biolegend, San Diego, CA, USA), and goat anti-mouse IgG polyclonal antibody (1:3,000, GTX213111-01; GeneTex, Irvine, CA, USA). Each experiment was repeated three times, and GAPDH mouse monoclonal antibody (1:1,000, 437,000; Invitrogen) was used as an internal reference protein. Finally, signals were detected by GE Healthcare’s ECL detection reagent (GE Healthcare, Chicago, IL, USA).

### RNA-Seq and real-time RT-PCR

Total RNA integrity was assessed using an Agilent 2100 biological analyzer (Agilent Technologies, Santa Clara, CA, USA). Samples with RNA integrity values of > 7.0 were used for RNA-Seq. The complementary DNA libraries were sequenced on the Illumina HiSeq X Ten platform. The differential expression was analyzed using the software DESeq (2012) R package. *P* < 0.05, fold-change > 2, and FDR < 0.01 were set as the thresholds for significant differential expression. Based on the hypergeometric distribution, R was used to enrich differentially expressed genes by Gene Ontology and KEGG, respectively. For quantification of gene expression, the 2^−ΔΔCT^ method was used and normalized against GAPDH expression. The primers of all primer sets used in these experiments are shown in Online Table 1.

### CUT&Tag

CUT&Tag for bench-top application was performed using 100,000 cells as previously published [81]. More detailed information can be found at the website: http://www.protocols.io/view/bench-top-cut-amp-tag-wnufdew/abstract. The primary antibody was H3K4me3 rabbit monoclonal antibody (1:500, 9751; CST), and the secondary antibody was goat anti-rabbit IgG (AS070; ABclonal, Wuhan, China). The DNAs were extracted and subjected to PCR. The primers of all primer sets used in these experiments are shown in Online Table 1. The experiments were repeated three times independently and the results were normalized to the IgG control.

### HC, SGN, and synapse counts

Quantification of HCs, SGNs, and synapses was performed in the apical, middle, and basal turns of the cochlea. For the evaluation of HC, we took 63 × magnified confocal images of each turn of the cochlea at random as representative images. We calculated the number of total HCs in the images, averaged the results for three images from each turn, and presented the data as per 200 µm. For cultured SGN explants, the fluorescence images were acquired using confocal laser microscopy with a 40 × oil immersion objective with a 0.75 × digital zoom and a 1 µm step size between each plane. The length of SGN neurites and the SGN density per 100 μm were calculated. For synaptic quantification, we obtained confocal z-stacks images of IHC area from each turn with a 63 × oil immersion objective with a 2 × digital zoom and a 1 µm step size between each plane. Z-stacks were allowed to span all IHCs, including all synaptic ribbons. We imported the image stacks into ImageJ (National Institutes of Health, Bethesda, MD, USA) and then manually calculated the CtBP2 or GluR2 puncta in each image stack. The number of CtBP2 or GluR2 puncta per IHC within a 100 μm range was calculated in three different regions of every turn of the cochlea.

### Statistical analyses

Data were analyzed and plotted by GraphPad Prism software (San Diego, CA, USA) and shown as mean ± SEM. Quantitative data statistical analysis was assessed using one-way analysis of variance (ANOVA) for multiple group comparisons or two-tailed Student's *t* tests for two-group data. *P* < 0.05 was considered as significant.

## Supplementary Information

Below is the link to the electronic supplementary material.

Supplementary file1 (DOCX 8142 KB)

## Data Availability

All data generated or analyzed during this study are included in this published article and its supplementary information files.

## Abbreviations

ABR: Auditory brainstem response
CP: Cisplatin
DEGs: Differentially expressed genes
GRPR: Gastrin-releasing peptide receptor
HCs: Hair cells
H&E: Hematoxylin and eosin
HIF-1: Hypoxia-inducible factor
IHCs: Inner hair cells
JmjC: Jumonji C
KDM5A: Lysine-specific demethylase 5A
KEGG: Kyoto Encyclopedia of Genes and Genomes
MMP: Mitochondrial membrane potential
OCRs: Oxygen consumption rates
OHCs: Outer hair cells
PI: Propidium iodide
ROS: Reactive oxygen species
SGNs: Spiral ganglion neurons
SOS: Son of sevenless
TUNEL: Terminal deoxynucleotidyl transferase-mediated dUTP nick-end-labeling
