# Supplementary material for: Inhibition of KDM5A attenuates cisplatin-induced hearing loss via regulation of the MAPK/AKT pathway
Source: Cell Mol Life Sci. 2022 Nov 17;79(12):596. doi: 10.1007/s00018-022-04565-y (PMC9672031; doi:10.1007/s00018-022-04565-y)
Supplement: Supplementary file 1 — Supplementary file1 (DOCX 8142 KB) [file 18_2022_4565_MOESM1_ESM.docx]

**Inhibition of KDM5A attenuates cisplatin-induced hearing loss via regulation of the MAPK/AKT pathway**

**Supplementary Material**

**
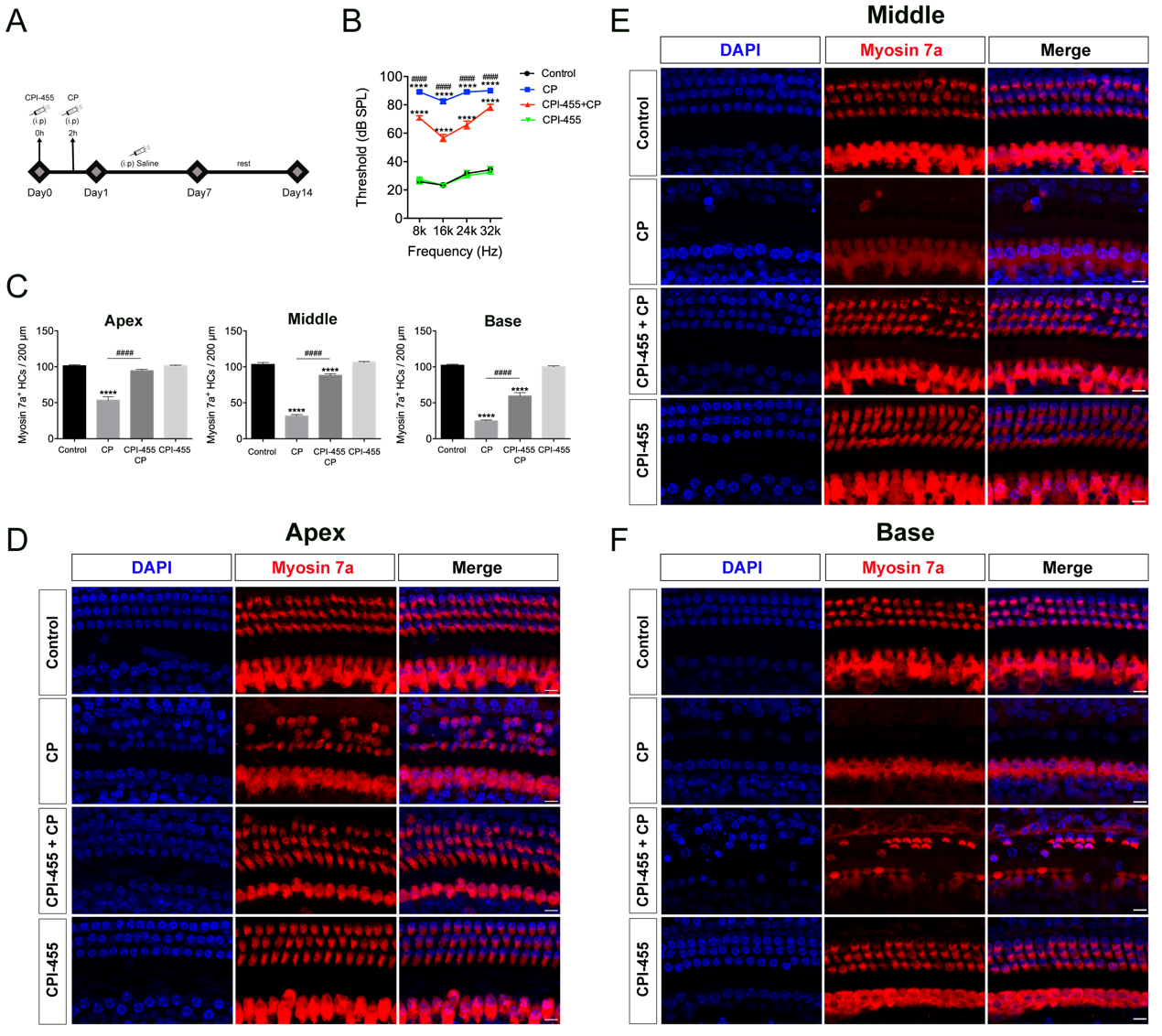
**

**Supplemental Figure 1**

Effects of CPI-455 on CP-induced hearing loss *in vivo*. **A** Experimental workflow. **B** ABR analysis. Data are shown as mean ± SEM values. *****P* < 0.0001 compared with the control; ^####^*P* < 0.0001 compared with CP only. *n* = 6 mice for each group. The experiments were performed in triplicate. **C** Graphical representation of the number of Myosin 7a–positive hair cells. The data are presented as mean ± SEM values. *****P* < 0.0001 compared with the control; ^####^*P* < 0.0001 compared with CP only. The experiments were performed in triplicate. *n* = 12 cochleae for control group; *n* = 12 cochleae for CP group; *n* = 12 cochleae for CPI-455 + CP group; *n* = 12 cochleae for CPI-455 group. **D–F** Cochleae were stained with Myosin 7a (red) and DAPI (blue). Scale bar = 10 µm.


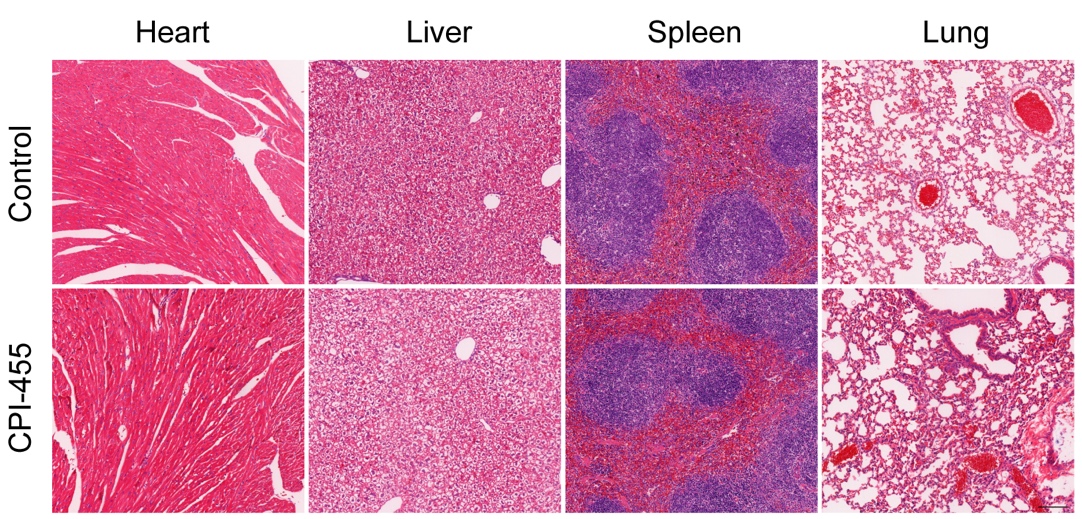


**Supplemental Figure 2**

Administration of CPI-455 exhibited no significant toxicity in the heart, liver, spleen, and lung organs. H&E staining was used to evaluate the histology. Bar = 100 μm.


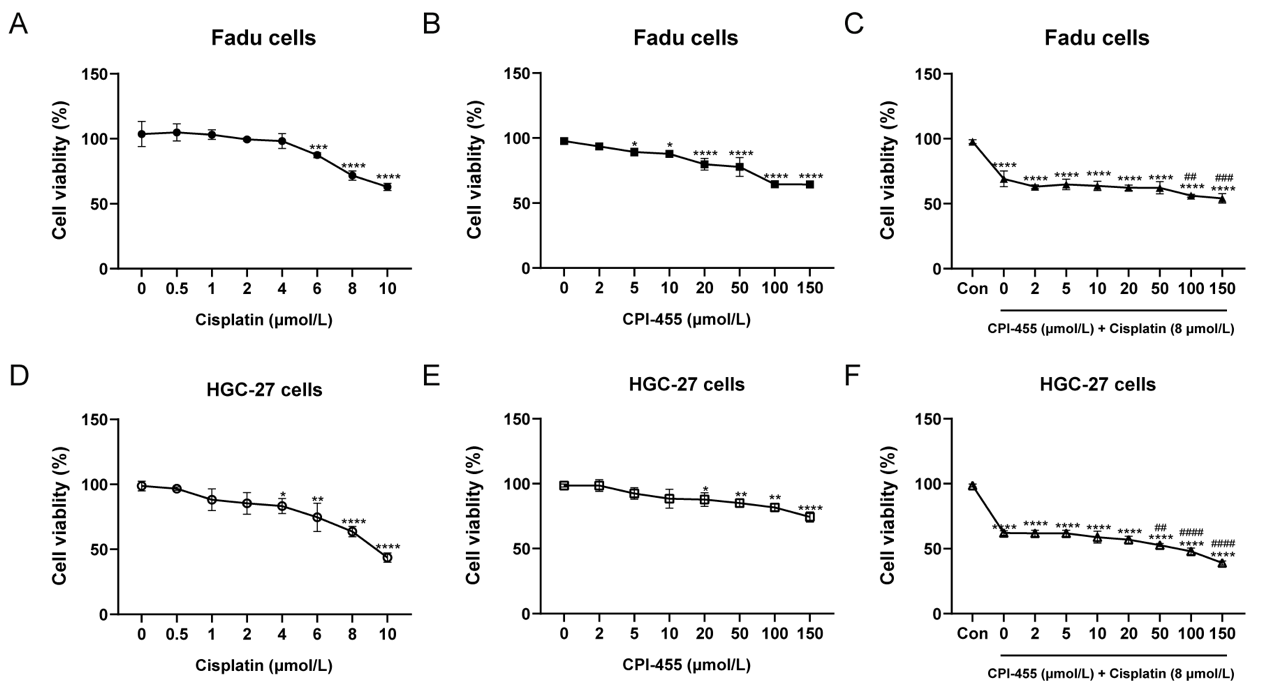


**Supplemental Figure 3**

The sensitivity of two tumor cell lines to CP and/or CPI-455. The two tumor cell lines were Fadu (head and neck cancer) and HGC-27 (gastric cancer) cells. Different sensitivities to CP (A, D), CPI-455 (B, E), and their combinations (C, F) were observed. The data are presented as three independent experiments' mean ± SEM values.

**
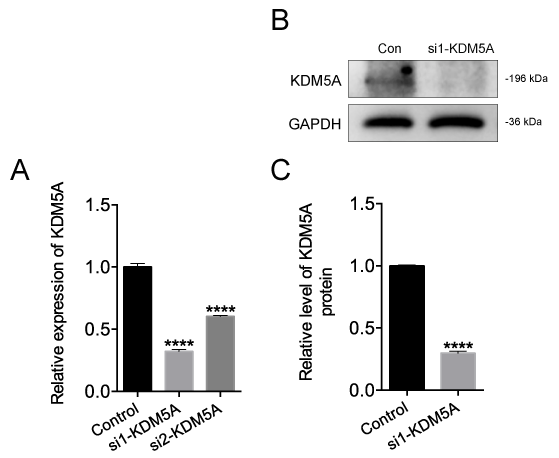
**

**Supplemental Figure 4**

**A** mRNA expression of KDM5A was detected by qRT-PCR. **B** KDM5A protein expression level was determined by western blot. Representative pictures are shown. **C** KDM5A protein-expression level was quantified in HEI-OC1 cells. Data are presented as mean ± SEM values. *****P* < 0.0001 compared with the control. The experiments were performed in triplicate.

| Symbol name | Primer sequence |  |
| --- | --- | --- |
| **For Real-Time PCR assay** |  |  |
| *Trp53* | 5’-CTCTCCCCCGCAAAAGAAAAA-3’ |  |
|  | 5’-CGGAACATCTCGAAGCGTTTA-3’ |  |
| *Bad* | 5’-AAGTCCGATCCCGGAATCC-3’ |  |
|  | 5’-GCTCACTCGGCTCAAACTCT-3’ |  |
| *Fos* | 5’-CGGGTTTCAACGCCGACTA-3’ |  |
|  | 5’-TTGGCACTAGAGACGGACAGA-3’ |  |
| *Jun* | 5’-CCTTCTACGACGATGCCCTC-3’ |  |
|  | 5’-GGTTCAAGGTCATGCTCTGTTT-3’ |  |
| *Gadd45b* | 5’-CAACGCGGTTCAGAAGATGC -3’ |  |
|  | 5’-GGTCCACATTCATCAGTTTGGC -3’ |  |
| *Nfe2l2* | 5’-TCTTGGAGTAAGTCGAGAAGTGT-3’ |  |
|  | 5’-GTTGAAACTGAGCGAAAAAGGC-3’ |  |
| *Pik3ca* | 5’-CCACGACCATCTTCGGGTG -3’ |  |
|  | 5’-ACGGAGGCATTCTAAAGTCACTA -3’ |  |
| *Sos1* | 5’-CAAGTTCACCCTACTCTTGAGTC-3’ |  |
|  | 5’-CATCAGCTATTGCCCACTTATCA-3’ |  |
| *Sos2* | 5’- CAAGATGTTGAGGAACGAGTTCA-3’ |  |
|  | 5’-TGTCCACAGGTAGTAAGAGAGGA-3’ |  |
| *Akt3* | 5’-TGGGTTCAGAAGAGGGGAGAA-3’ |  |
|  | 5’-AGGGGATAAGGTAAGTCCACATC-3’ |  |
| *Map3k3* | 5’-ATAAGGACACAGGTCACCCAA-3’ |  |
|  | 5’-TGCTCCACATCTTCGTATCTCA-3’ |  |
| *Map3k1* | 5’-CGCCCTGCCCATCTACTTC-3’ |  |
|  | 5’-CTCCATCTCTCGACCGGAGG-3’ |  |
| *Mapk8* | 5’-AGCAGAAGCAAACGTGACAAC-3’ |  |
|  | 5’-GCTGCACACACTATTCCTTGAG-3’ |  |
| *Gapdh* | 5’-AGGTCGGTGTGAACGGATTTG-3’ |  |
|  | 5’-TGTAGACCATGTAGTTGAGGTCA-3’ |  |
| *Kdm5a* | 5’-CACAGACCCGCTGAGTTTTAT-3’  5’-CTTCACAGGCAAATGGAGGTT-3’ |  |
| **For Cut&Taq-qPCR assay** |  |  |
| *Sos1* (-1000~-501) | 5’-AGCGTTCAATGATAGAGCACA-3’ |  |
|  | 5’-ACTTGCTTCCTGTAGTGGCA-3’ |  |
| *Sos1* (-1500~-1001) | 5’-GAGATTGGCCTTGCTCCTCT-3’ |  |
|  | 5’-TCAAGCTGCGAGAAGTGATG-3’ |  |
| *Sos2* (-1000~-501) | 5’-CCTTCAACGTGCGCTAAGAG-3’ |  |
|  | 5’-TTAACGTGTGCTCTGTGTGG-3’ |  |
| *Sos2* (-1500~-1001) | 5’-CGGTTCTTTCCTTCACGTGG-3’ |  |
|  | 5’-GGAAAAGGTACCTGCTTCGC-3’ |  |
| *Map3k3(*-1500~-1001) | 5’-CAAACCTTCCCTGCCCAATC-3’ |  |
|  | 5’-TATGGTTGTCGGTTGTTGGC-3’ |  |
|  |  |  |

**Table 1.** All the primers used in this study are listed.
